# Primary bronchial schwannoma: A case report

**DOI:** 10.1097/MD.0000000000031062

**Published:** 2022-10-07

**Authors:** Yosuke Aoyama, Atsushi Miyamoto, Takeshi Fujii, Sakashi Fujimori, Meiyo Tamaoka, Daiya Takai

**Affiliations:** a Department of Respiratory Medicine, Respiratory Center, Toranomon Hospital, Tokyo, Japan; b Department of Pathology, Toranomon Hospital, Tokyo, Japan; c Department of Thoracic Surgery, Respiratory Center, Toranomon Hospital, Tokyo, Japan.

**Keywords:** bronchial neoplasms, bronchoscopy, neurinoma, Schwannoma, thoracic surgery

## Abstract

Bronchial schwannomas are extremely rare among the benign tracheobronchial tumors and little are known about its epidemiology and optimal clinical management. Here, we report a case of bronchial schwannoma in a young Japanese man and clinical implications about epidemiology, symptom, diagnosis, and treatment of bronchial schwannoma.

**Patients concern:**

A 37-year-old man visited our department with a nodule incidentally found on his chest radiograph during a routine medical checkup.

**Diagnosis:**

The tumor was diagnosed as a bronchial schwannoma after pathological evaluation. Microscopically, the tumor consisted of spindle cell proliferation characterized by an alternating highly ordered cellular Antoni A component with occasional nuclear palisading and a loose myxoid Antoni B component. Tumor cells were immunoreactive for S100 but not for smooth muscle actin or KIT.

**Interventions:**

A video-assisted right middle and lower bilobectomy was performed.

**Outcome:**

He remains under observation without recurrence.

**Lessons:**

In our review, many reports have come from Asian countries. Bronchial schwannoma can occur within a wide range of age groups and in both men and women. No difference in incidence was observed between right and left bronchial tree. Bronchial schwannoma is sometimes difficult to differentiate from malignant diseases. We should include bronchial schwannoma as one of the differential diagnoses of primary bronchial tumors.

## 1. Introduction

Schwannoma is a benign encapsulated nerve sheath tumor arising in peripheral nerves. Bronchial schwannomas are extremely rare and account for 2.2% of benign tracheobronchial tumors.^[1,2]^ Due to its rarity, the clinical detail of this tumor including clinical presentation, imaging features, and standard therapy, have been insufficiently reported. In addition to presentation of our case, we reviewed published cases of bronchial schwannomas, evaluated their epidemiology, and tried to clarify these issues.

## 2. Patient information

A 37-year-old Japanese man visited our hospital after a nodule was found on chest radiograph during a routine medical checkup. He was asymptomatic and had no past medical history and no family history of malignant disease within the second degree of kinship. He was an office worker with no smoking or dust exposure history.

## 3. Clinical findings

Vital signs, including body temperature and oxygen saturation, were normal. No wheeze or crackle was heard on auscultation. No lymph nodes were palpable.

## 4. Diagnostic assessment

**Figure 1.**
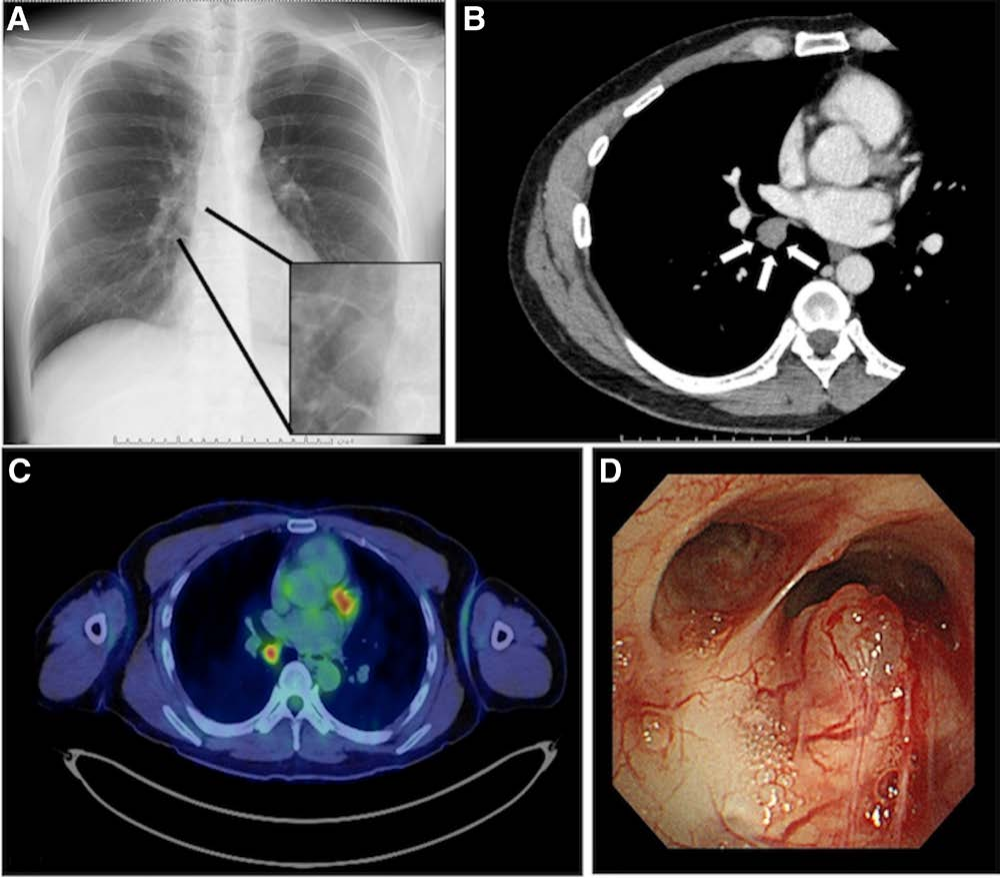
(a) A nodule is noted on the chest radiograph (black arrows). (b) A 17-mm-sized nodule, with a mild contrast effect, is noted on contrast-enhanced CT (white arrows). (c) 18F-fluorodeoxyglucose (FDG) positron emission tomography/computed tomography (PET-CT) reveals abnormal FDG accumulation in the nodule, with a maximum standardized uptake value of 4.07. (d) Bronchoscopy reveals a non-pulsating submucosal nodule, with proliferating capillary vessels, on the mucosal surface of the proximal end of the right intermediate bronchus.

Milestones related to diagnosis and intervention is shown in Table 1. Laboratory investigation results, including tumor markers, were unremarkable. Chest radiograph revealed a discrete nodule located at the caudal part of the right hilum (Fig. 1a). Computed tomography revealed a 17-mm-sized nodule in the right lower lobe (Fig. 1b). 18F-fluorodeoxyglucose (FDG) positron emission tomography/computed tomography (PET/CT) revealed abnormal FDG accumulation in the nodule, with 4.07 times the maximum standardized uptake values (Fig. 1c). Bronchoscopy identified a non-pulsating submucosal nodule with proliferating capillaries on the mucosa at the proximal end of the right intermediate bronchus (Fig. 1d). The nodule bled easily upon contact.

**Table 1:** Milestones related to diagnosis and intervention.

1 yr before the first visit to our department	No abnormality was detected on his chest radiograph
3 wk before the first visit	A nodular opacity was found on his chest radiograph
2 wk after the first visit	Computed tomography revealed a nodule in the right lower lobe
5 wk after the first visit	FDG-PET/CT revealed abnormal FDG accumulation in the nodule. A submucosal nodule was identified via bronchoscopy
7 wk after the first visit	A video-assisted right middle and lower bilobectomy was performed

CT = computed tomography, FDG = 18F-fluorodeoxyglucose, PET = positron emission tomography.

## 5. Therapeutic intervention

**Figure 2.**
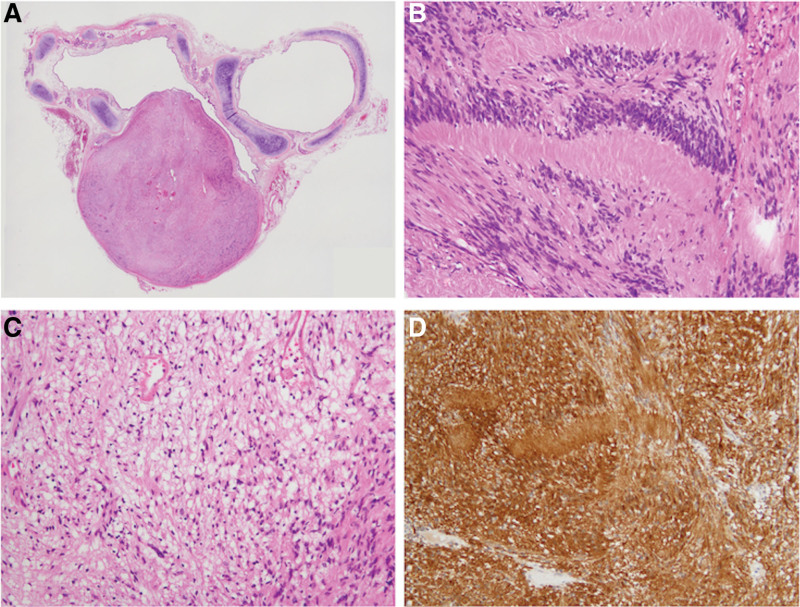
(a) Loupe view. A dumbbell-shaped nodule, measuring 20 × 15 × 15 mm in dimension, appears to be localized within the bronchial wall, limited by the bronchial adventitia, and protruding between the cricoid cartilages into the lumen. (b–d) Histological and immunohistochemical findings. (b, c) hematoxylin and eosin stains. The tumor consists of spindle cell proliferation characterized by an alternating highly ordered cellular Antoni A component (b) with occasional nuclear palisading and a loose myxoid Antoni B component (c). Tumor cells were immunoreactive to S100 (d). The nodule was histologically diagnosed as a schwannoma.

A video-assisted right middle and lower bilobectomy was performed. Gross examination of the nodule showed a dumbbell-shaped solid tumor surrounded by a fibrous capsule with a mottled yellow-white appearance on the cut section. A loupe view is shown in Figure 2a. Microscopically, the tumor consisted of spindle cell proliferation characterized by an alternating highly ordered cellular Antoni A component (Fig. 2b) with occasional nuclear palisading and a loose myxoid Antoni B component (Fig. 2c). Tumor cells were immunoreactive for S100 (Fig. 2d) but not for smooth muscle actin or KIT. The tumor was localized within the bronchial wall, limited by the bronchial adventitia, and protruding between the cricoid cartilages into the lumen. The tumor was diagnosed as a bronchial schwannoma.

## 6. Follow-up and outcomes

The patient had a good postoperative course and remains under observation in an outpatient clinic. Image findings show that the tumor has not recurred to date.

## 7. Discussion

**Figure 3.**
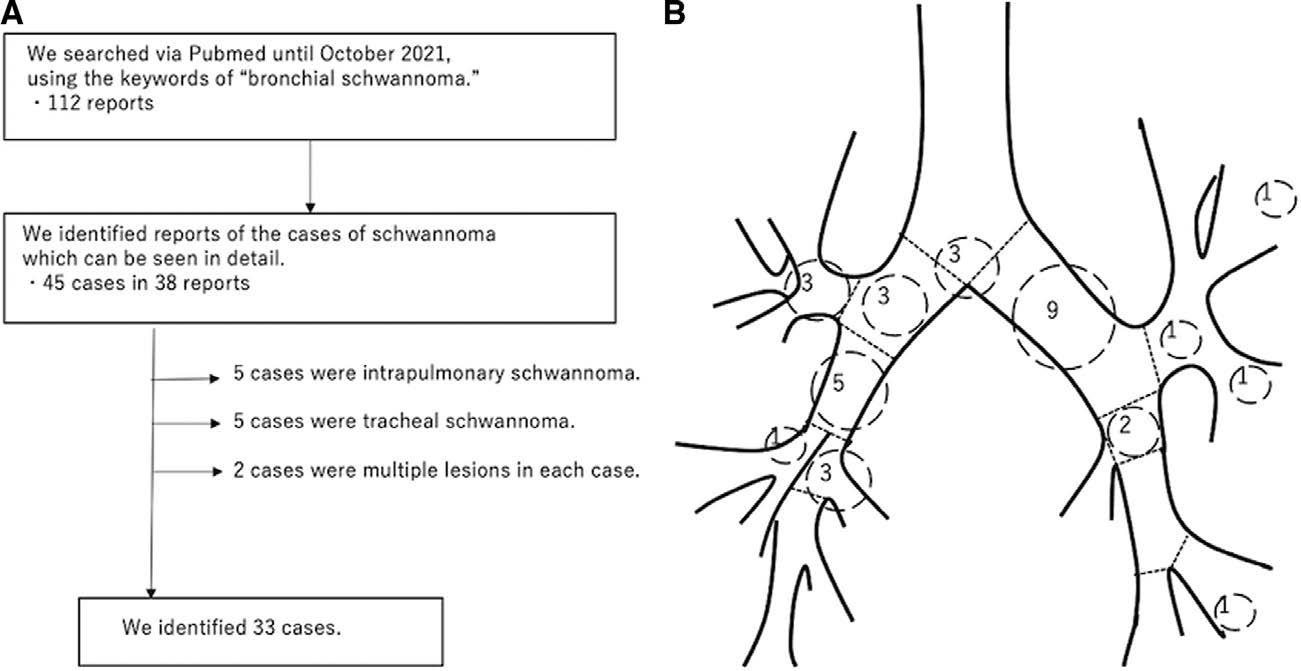
(a) The review process of previously reported cases of bronchial schwannomas. (b) The number of schwannomas encountered per bronchial location is tallied from the previously reported cases. Three schwannomas were in the carina, three in the right main bronchus, three in the right upper bronchus, five in the intermediate bronchus, one in the right middle bronchus, three in the right lower bronchus, nine in the left main bronchus, three in left upper bronchus area, and three in left lower bronchus area.

We searched the cases with the keyword “bronchial schwannoma” via PubMed in October 2021 and identified 112 articles. We identified 33 cases of bronchial schwannoma among them (Table 2).^[3–28]^ The process of selecting the cases is shown in Figure 3a. The median age was 48 years. Of 33 cases, 16 were male and 17 were female. In terms of tumor location, 15 were in the right bronchial tree, 15 were in the left bronchial tree and 3 were in the trachea or carina (Fig. 3b). This review indicates that bronchial schwannoma occurs not only in older people but also in younger people, with no apparent differences with regards to gender or tumor location. Although many reports have come from Asian countries, we cannot definitively state the frequency of occurrence based on race.

**Table 2:** Compilation of data from previous studies^[3–28]^

The number of reported cases	N = 33
Median age (range)	48 yr (13–86)
Sex (male: female)	16: 17
Country	
Asian countries (Japan/others)	N = 29 (N = 18/N = 11)
Western countries	N = 4
Tumor origin	
Carina	N = 3
Right side	N = 15
Left side	N = 15

Bronchial schwannomas are often detected and diagnosed with clinical presentations of cough, hemoptysis, and dyspnea; and some cases are diagnosed after the development of obstructive pneumonia.^[8,29]^ However, cases have been reported in which bronchial schwannomas were found incidentally through bronchoscopy in patients without symptoms.^[18]^ Moreover, some cases are discovered on chest radiographs during medical checkups, such as this case.^[8]^ Although FDG-PET/CT is helpful in distinguishing between benign and malignant lung tumors, maximum standardized uptake values on FDG-PET/CT of schwannomas has been shown to be varied, probably due to the degrees of cellularity, microvascular density or vascular permeability.^[30,31]^ It should be noted that FDG-PET/CT is not always practical in differentiating a schwannoma from a malignant tumor, as a schwannoma sometimes shows abnormal FDG accumulation, as was the case in this patient. Histological evaluation is the standard method used for the diagnosis of schwannoma. The presence of typical Antoni A and Antoni B patterns and expression of S100 protein helps the pathological diagnosis of schwannoma.^[8,18,29]^ Surgical resection or resection by less-invasive techniques is the usual treatment for bronchial schwannomas.^[29]^ Resection with bronchoscopy has been attempted instead of surgical resection.^[8,32]^ The tumor should be resected completely to avoid local recurrence due to incomplete resection.^[18]^ In this case, we could not confirm the diagnosis before surgical resection as the tumor bled easily and therefore could not be biopsied through bronchoscopy. The results from FDG-PET/CT could not rule out a malignant tumor. Without resection, the growing tumor was expected to cause obstructive pneumonia as prior radiographs had not been reported to demonstrate a nodule.

In conclusion, bronchial schwannoma is a rare tumor but should be included as one of the differential diagnoses of primary bronchial tumors. The epidemiology of bronchial schwannomas is not fully understood, but they can occur within a wide range of ages and in both men and women. Accurate examination, diagnosis, and treatment should be made correctly. Further investigation and accumulation of cases is needed.

## 8. Patient perspective

The patient achieved histopathological confident diagnosis of the tumor and to rule out the possibility that it might be malignant tumor. The accurate diagnosis and successful treatment relieved the patient of developing additional difficult comorbidities including obstructive pneumonia or lobar atelectasis.

## Author contributions

**Conceptualization:** Yosuke Aoyama, Atsushi Miyamoto, Takeshi Fujii, Sakashi Fujimori, Meiyo Tamaoka, Daiya Takai.

**Data curation:** Yosuke Aoyama, Takeshi Fujii.

**Formal analysis:** Yosuke Aoyama, Takeshi Fujii.

**Investigation:** Yosuke Aoyama, Atsushi Miyamoto, Takeshi Fujii, Sakashi Fujimori.

**Methodology:** Yosuke Aoyama, Atsushi Miyamoto, Sakashi Fujimori, Meiyo Tamaoka, Daiya Takai.

**Project administration:** Atsushi Miyamoto, Takeshi Fujii, Sakashi Fujimori, Meiyo Tamaoka, Daiya Takai.

**Resources:** Sakashi Fujimori.

**Supervision:** Atsushi Miyamoto, Takeshi Fujii, Sakashi Fujimori, Meiyo Tamaoka, Daiya Takai.

**Validation:** Yosuke Aoyama, Takeshi Fujii.

**Visualization:** Yosuke Aoyama, Takeshi Fujii.

**Writing – original draft:** Yosuke Aoyama.

**Writing – review & editing:** Yosuke Aoyama, Atsushi Miyamoto, Takeshi Fujii, Sakashi Fujimori, Meiyo Tamaoka, Daiya Takai.
